# Practices and preferences in the use of magnification among endodontists and restorative dentists: A multicentre study

**DOI:** 10.1371/journal.pone.0311391

**Published:** 2025-01-07

**Authors:** Azhar Iqbal, Mohmed Isaqali Karobari, Deepti Shrivastava, Kumar Chandan Srivastava, Bilal Arjumand, Hmoud Ali Algarni, Meshal Aber Alonazi, Muhsen Alnasser, Osama Khattak, Jamaluddin Syed, Reham Mohmad Attia, Asma Abubakar Rashed, Sherif El Sayed sultan

**Affiliations:** 1 Department of Restorative Dentistry, College of Dentistry, Jouf University, Sakaka, Saudi Arabia; 2 Department of Conservative Dentistry and Endodontics, Saveetha Dental College and Hospital, Saveetha Institute of Medical and Technical Sciences, Saveetha University, Chennai, Tamil Nadu, India; 3 Department of Preventive Dentistry, College of Dentistry, Jouf University, Sakaka, Saudi Arabia; 4 Department of Oral & Maxillofacial Surgery & Diagnostic Sciences, College of Dentistry, Jouf University, Sakaka, Saudi Arabia; 5 Department of Oral Medicine & Radiology, Saveetha Institute of Medical and Technical Sciences, Saveetha Dental College, Saveetha University, Chennai, Tamil Nadu, India; 6 Department of Conservative Dental Sciences and Endodontics, College of Dentistry, Qassim University, Buraydah, Saudi Arabia; 7 Researcher and Visiting Consultant, Pioneer Public Health Consultants, Houston, Texas, United States of America; 8 Top Blood Laboratories Sugarland, Sugar Land, Texas, United States of America; 9 Conservative Dentistry Department, Faculty of Dentistry, Zagazig, Egypt; 10 Restorative Dentistry Department, Tanta University, Tanta, Egypt; 11 Department of Prosthetic Dental Sciences, Jouf University, Sakaka, KSA Kingdom of Saudi Arabia; 12 Department of Fixed Prosthodontics, Tanta University, Tanta, Egypt; International Medical University, MALAYSIA

## Abstract

**Background and objectives:**

Aim of the current study was to assess the perception, preference, and practice of endodontists and restorative dentists at different locations around the world about dental magnification instruments.

**Materials and methods:**

A multicenter, cross-sectional study was ethically approved from the local committee of bioethics. After thorough literature search, a questionnaire was designed and validated. Later, the questionnaire was distributed to 10% (53 participants) of the total planned participants to conduct a pilot study. Based on the feedback from these participants, any ambiguities or discrepancies observed in the items and content of the questionnaire was modified. The questionnaire was assessed for its internal consistency as part of validating the items with Cronbach’s alpha of 0.80. The completed questionnaire with an informed consent form for the participant was administered to the endodontists and restorative dentists in three different geographical regions namely MENA (Middle East and Northern Africa), British-Isles, and Indian Sub-continent using WhatsApp through the snowball convenience sampling technique.

**Results:**

Majority of the participants were male (56.5%) and in the age group of 25–35 years (30.3%). About 68.9% were from Indian sub-continent, followed by the British-Isles (16.5%) and the least (14.6%) were from the MENA region. By large, the participants of the present study, strongly agreed that dental magnification devices improved ergonomics, quality of work, and should be considered as standard of care in modern endodontic. Flip-up magnifiers (51.1%) and medium (8x-16x) magnification were preferred by majority of the participants. About 46.3% of specialist reported that they always used devices for all operative and endodontic procedures, especially while locating hidden and canals and negotiating calcified canals. Participants practicing in British-Isles have 2.42 times (P<0.05) higher adequate perception with reference participants in Indian sub-continent. Additionally, participants with fellowship have 2.77 times more (P<0.01) adequate perception with reference to their counterparts with a master’s degree.

**Conclusions:**

Most of the participants believe that dental magnification devices enhance the prognosis and quality of treatment of possibly all operative and endodontics procedures. Thus, emphasized on the inclusion of devices in the postgraduate curriculum and signifies the role of continuing dental education for specialist and dental assistant handling devices. However, multicenter studies with larger sample is required for generalizing the results.

## 1. Introduction

Before the advent of dental magnification, dentists were relying on their tactile sensitivity and radiographs while performing various dental procedures including endodontic and restorative dentistry. There was an increased chance of procedural errors and that were mostly dealt with patience and dental imaging [1]. Over the past years to overcome these procedural errors, many magnification devices were introduced as bridging tools between the human naked eyes and the microscope [2]. However, the tools such as an endoscope, magnifying glass, and intraoral camera were largely superseded by contemporary devices that seems to be more practical and convenient for application, such as dental loupes and dental operating microscope (DOM) [1, 3].

The necessity of enhanced visualization during the clinical work mandates the endodontists and restorative dentists to adopt dental magnification in their routine dental practice. The use of dental magnification has proven to improve the visualization and ergonomics benefits for the clinicians including an improved posture with reduced musculoskeletal stress [2–4]. Nowadays magnification devices are being used for simple and complicated dental procedures such as caries removal, scaling and polishing, preparation of crown margins, location of hidden canals, perforation and furcation repair, removal or placement of a variety of posts, endodontic surgical procedures, and bone and soft tissue grafting procedures [5].

Recent literature supports the use of dental magnification tools namely dental operating microscope (DOM) and loupes to enhance the clinical outcomes significantly especially for procedures that require visual enhancement of the surgical fields in restorative dentistry and endodontics [6, 7]. In the United States and United Kingdom, the dentists have demonstrated the increasing use of dental magnification devices [8, 9], but on the other hand, Turkish dentists (85.5%) serving in the public sector institutions remained reluctant and resistant to practice dental magnification tools [10].

Few studies in the literature demonstrate consistent outcomes when magnification is integrated into the clinical setting, despite the paucity of scientific evidence regarding the impact of magnification on the dentist’s performance. [11]. Hence, the present questionnaire-based, multicentre, cross-sectional study was conducted to evaluate the perceptions, preferences, and practices of endodontists and restorative dentists towards the use of dental magnification armamentarium.

## 2. Materials and methods

### 2.1 Ethical considerations

A multicentre, explorative, cross-sectional study was designed. It was ethically approved from the local committee of bioethics at King Abdulaziz University, Jeddah, Kingdom of Saudi Arabia (029-02-22A).

### 2.2 Study tool

#### Preparation of the questionnaire

Initially an exhaustive search of the published research with similar objective and methodologies was carried out. Later, the group of selected articles were thoroughly reviewed and eventually three articles were segregated. Based on the aim and objective of the current study, questionnaires from the selected studies were retrieved and electronic consent was taken from the respective corresponding authors [11–13]. Considering the objectives of the current study, nature of study population, and local factors, a team of 3 members that comprised of endodontist, restorative dentist, and a biostatistician prepared a modified version of questionnaire for the current study. After repeated revisions of the contents, the questionnaire was organized and structured. Later, the structured questionnaire was tested in a pilot study by distributing it among a small group of about 53 participants. Considering the feedback received from the pilot study, any ambiguity or discrepancy observed in the items and contents of the questionnaire was modified by the team. The questionnaire was assessed for its internal consistency by a biostatistician as a part of validating the items, with Cronbach’s alpha of 0.80.

#### Components of the questionnaire

The questionnaire had four sections with a total of 29 questions. The first section had seven questions to record the demographic information of the participants. The second section composed of 15 closed ended questions to assess the perceptions of the participants about the dental magnification. Out of 15 questions, only three questions namely no. 8–10 was negatively worded. A 5-Point Likert scale (strongly agree = 5) was used to assess the perception of the participants about the dental magnification. The third and fourth segments of the questionnaire were composed of 4 and 3 closed ended questions respectively. They were intended to evaluate about the preferences and practices of the participants about the dental magnification armamentarium in their daily practices.

### 2.3 Study population and sample size calculation

The study population included male and female endodontists and restorative dentists in the age range between 25 to 65 years from three different geographical regions namely MENA (Middle East and Northern Africa), British-Isles, and Indian Sub-continent. All endodontists and restorative dentists having completed their postgraduate education in endodontics and restorative dentistry were considered in the study. The participants that were engaged in academics, academics with clinical practice, and clinical practice alone in a public sector or private institutions, were included in the present study. Endodontist and restorative dentist who are currently not in active practice, or specialist who did not give consent to participate were excluded from the study. Additionally, the participants of the pilot study were also excluded. The sample size was calculated using Creative Research Systems sample size calculator (https://www.surveysystem.com/sscalc.htm) with Type 1 error rate set at 0.05 and power of study at 85%. A total of 534 participants were required to complete the analysis of present study. The sample size has been increased by more than 10% to account for potential non-responses due to participant ineligibility and incomplete responses [14]. Rasch model analysis was used to distinguish any disparity among the participants from different nations [15].

### 2.4 Study timeframe and study protocol

The data was collected between 1^st^ March 2022 to 31^st^ May 2022. The final version of questionnaire with a statement of consent for the participant was circulated through dedicated WhatsApp groups having restorative and endodontic specialist as participants. These forms were sent in three different geographical regions namely MENA (Middle East and Northern Africa), British-Isles, and Indian sub-continent through snowball convenience sampling technique. After opening the survey link, the participant read the consent statement. Only if he/she agreed to the consent, participant got the access to the questionnaire. In otherwise situation where the potential participant had chosen to decline the consent, the survey got terminated. Any queries related to the questionnaire items were addressed on email and WhatsApp. The forms were individually scanned by the researchers to identify any missing data.

### 2.5 Data management

The response to all questions regarding the perception about dental magnification were gathered on a 5-point Likert scale; However, each question had a correct response. Accordingly, prior to data analysis, the responses were analysed and were marked as “correct” or “incorrect”. For the sake of data analysis, the responses were assigned with a dummy code of “1” for correct and “0” for an incorrect response. Overall score for each respondent was calculated by adding the score attained by each question. Eventually, the overall scores were categorized into 2 scales namely “adequate” and “inadequate” using the equation mentioned below.


$$\text{Class}\;\text{width}\;\left(\text{CW}\right)=\frac{\text{Maximum}\;\text{Value}-\text{Minimum}\;\text{Value}}{\text{Number}\;\text{of}\;\text{required}\;\text{class}\;\text{interval}}$$ (1)

Where, 15 is the maximum possible score and 0 is the minimum possible score and two class intervals are required. Accordingly, the overall score ranging from 0–8 are considered as “inadequate perception”, while overall score ranging from 9–15 are labelled as “adequate perception.”

### 2.6 Data analysis

The obtained data was entered in the excel sheet and later it was analysed with Statistical Package for the Social Sciences Software version 23.0 for Windows (SPSS Inc., Chicago, IL, USA). Descriptive analysis was done using numbers and percentages to define the different characteristics of the variables. To establish relationship between the different groups and categorical variables, the chi-square test or Fisher’s test were performed. The statistical significance was recognized when the P value was set at P < 0.05.

The Endodontists and Restorative scale were analysed using two-parameter logistic item response theory (2-PL IRT) analysis with the ltm package version 1.0.0 due to the scale’s unidimensionality and responses’ dichotomous output as either correct answer or incorrect answer. The cutoff value for the psychometric properties’ evaluation of the domain will be in the range of difficulty (-3 to +3) and discrimination (0.25 to infinity). The chi-square goodness-of-fit for each item was used to assess item fit, and modified parallel analysis was used to examine unidimensionality.

## 3. Results

**Fig 1:**
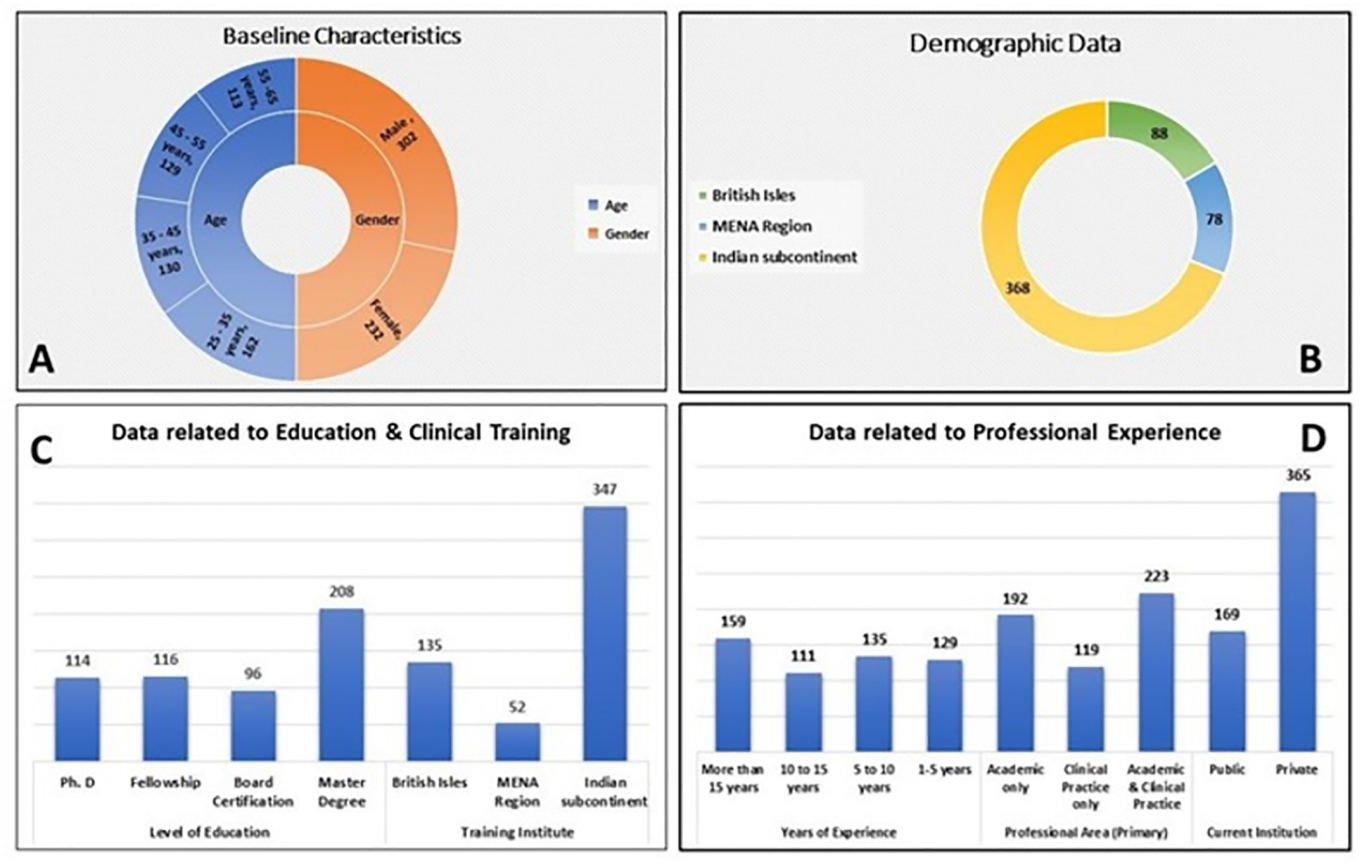
Baseline characteristics of the participants. (A)–Pie chart showing the age and gender distribution of the sample; (B)–Pie chart showing the distribution of geographical location of current job; (C)–Bar graph showing the highest level of education acquired; (D)- Bar graph showing the distribution of number of years of clinical experience while using dental magnification, Primary nature of clinical practice and Type of place of organization.

A total of 545 responses were gathered, out of which 12 responses were excluded from the study due to incomplete response. Accordingly, 534 participants were included in the current study. Fig 1A–1D shows the descriptive analysis of the baseline characteristics of the participants. Majority of the participants were male (56.5%) in the age group of 25–35 years (30.3%). Around 68.9% of participants were from Indian sub-continent, followed by British-Isles (16.5%) and then MENA region (14.6%). Participants with master’s degree were highest (39%) in the current survey and about 64.9% had undergone training from Indian sub-continent. Most of the study participants had more than 15 years of experience with about 42% engaged in academic as well as clinical practice. Largely, the participants were employed in private sector.

Majority of the participants strongly agreed that dental magnification devices will enhance the ergonomics (58.8%) by improving the vision (71%), preventing the eye fatigue (53.6%) and decreases the operating time (63.5%). Thus, it improves the prognosis (62%) and eventually the quality of work (57.1%). The participants believe that it can be considered as the standard of care in modern endodontic (60.9%). Hence, it should be made an essential component in the postgraduate training and curriculum (60.3%) and continuing dental education (39.3%). The participants responded in favour to the questions posed regarding the time taken to position the device and its maintenance (Table 1).

**Table 1:** Descriptive analysis of responses of participants about the perceptions towards using dental magnification devices.

S. No	Questions related to the perceptions towards using dental magnification devices	*Response n (%)*				
		*Strongly Disagree*	*Disagree*	*Neutral*	*Agree*	*Strongly Agree*
PQ1	Dental magnification devices will improve the **vision** of operator	1 (0.2)	3 (0.6)	20 (3.7)	131 (24.5)	379 (71.0)
PQ2	Dental magnification devices will prevent **eye fatigue** / strain of operator	1 (0.2)	15 (2.8)	65 (12.2)	167 (31.3)	286 (53.6)
PQ3	Dental magnification devices will improve the **ergonomics**	1 (0.2)	1 (0.2)	51 (9.6)	167 (31.3)	314 (58.8)
PQ4	Dental magnification will enhance the **quality of work**	9 (1.7)	22 (4.1)	42 (7.9)	156 (29.2)	305 (57.1)
PQ5	Dental magnification devices will make the treatment of **complicated cases** easy	3 0.6)	17 (3.2)	35 (6.6)	158 (29.6)	321 (60.1)
PQ6	Dental magnification devices will result in the **better prognosis** of endodontic retreatment and surgical endodontic cases	0	4 (0.7)	40 (7.5)	159 (29.8)	331 (62)
PQ7	Dental magnification devices are considered **standard of care** in modern Endodontic and Restorative dentistry practice	0	2 (.4)	36 (6.7)	171 (32.0)	325 (60.9)
PQ8	Takes too much time to **position the patient and specialist** for the dental magnification devices	36 (6.7)	216 (40.4)	162 (30.3)	97 (18.2)	23 (4.3)
PQ9	Difficult to **position the dental magnification devices** during procedures	14 (2.6)	163 (30.5)	139 (26)	160 (30)	58 (10.9)
PQ10	**Maintenance** of dental magnification devices is difficult	1 (0.2)	73 (13.5)	252 (47.2)	159 (29.8)	50 (9.4)
PQ11	Dental magnification devices will decrease the **operating time**	12 (2.2)	28 (5.2)	32 (6)	123 (23)	339 (63.5)
PQ12	It Should be an essential component of Postgraduate **training and curriculum** in Endodontic and Restorative Dentistry	0	47 (8.8)	72 (13.5)	93 (17.4)	322 (60.3)
PQ13	**CPD training** courses and workshops needed	28 (5.2)	19 (3.6)	131 (24.5)	156 (29.2)	200 (37.5)
PQ14	Special **training** needed for dental assistant for the disinfection and maintenance of dental magnification devices	43 (8.1)	318 (59.6)	108 (20.2)	39 (7.3)	26 (4.9)
PQ15	**Cost-effective** to use the dental magnification devices	180 (33.7)	128 (24)	162 (30.3)	64 (12.0)	0

**Fig 2:**
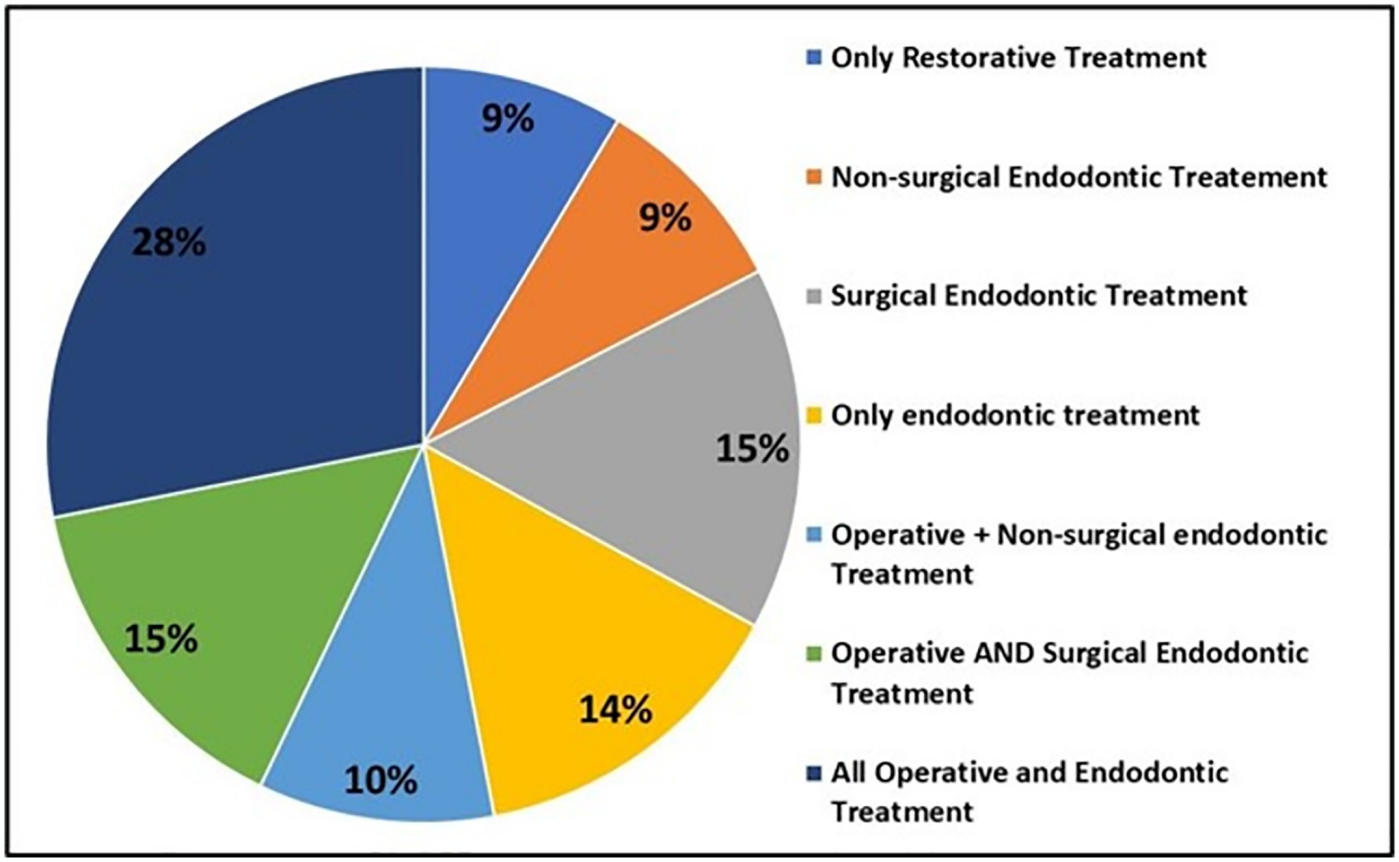
Pie chart showing the preferences of participants for using dental magnification devices in dental procedures.

**Fig 3:**
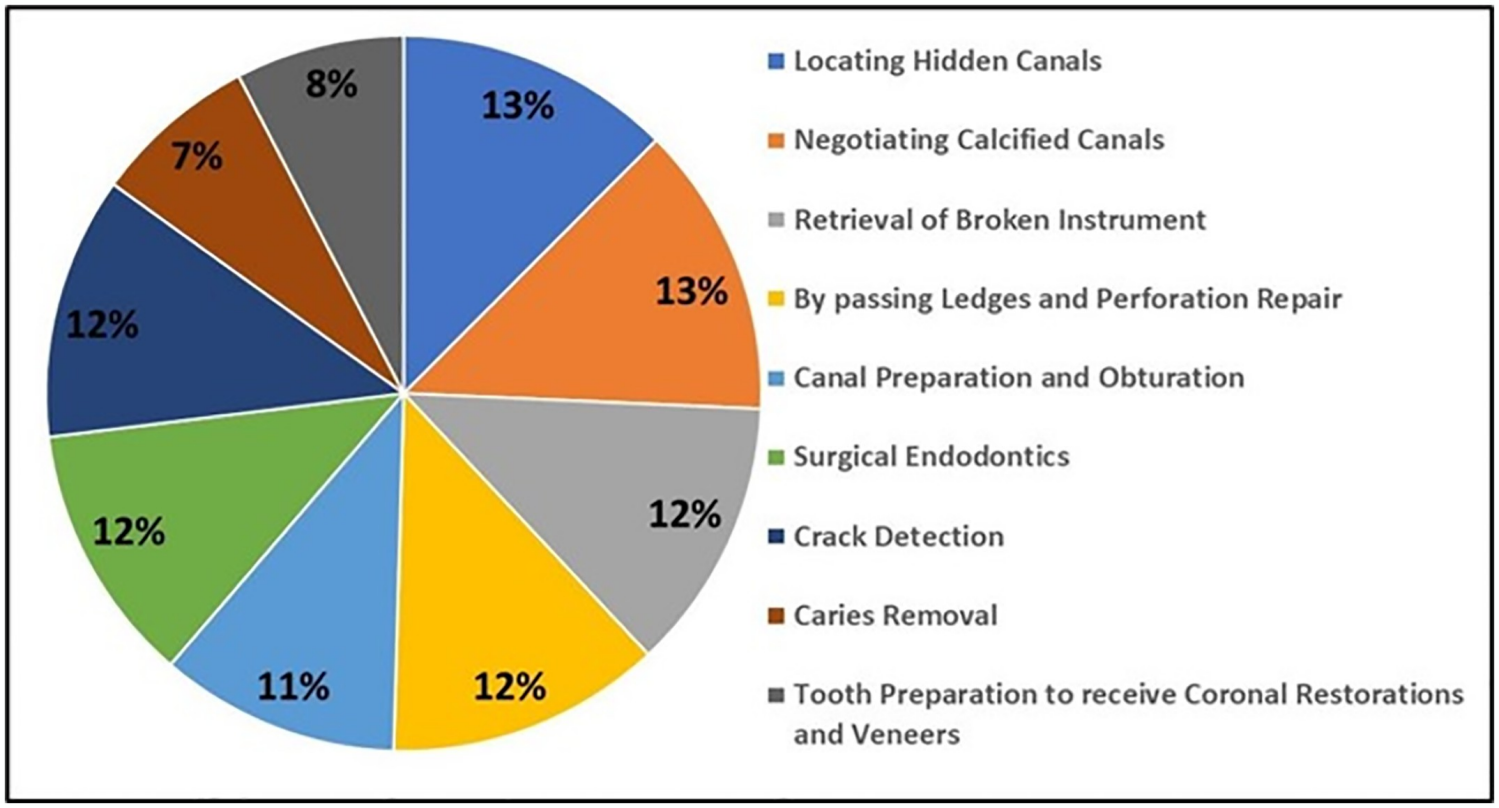
Pie chart showing the nature of procedures for which the dental magnification devices are used by the participants of the study.

The participating specialist reported to prefer flip up loupes (51.1%) followed by dental operating microscopes (DOM) (32.2%) along with medium level of magnification 48.5%. Majority (41.8%) reported to prefer delaying the procedure in case of absence of the dental magnification devices, thus showing inclination towards dental magnification devices (Table 2). Furthermore, magnification devices were found be used in all nature of operative and endodontics procedures (28.1%) (Fig 2). Locating hidden canal, negating calcified canals, retrieving broken instruments, bypassing ledges, surgical endodontic cases and crack detection were some of the reported procedures where devices are used routinely (Fig 3). Most of the participating specialist believed that magnification devices should be a part of postgraduate training program and continuing dental education programs will add further benefit (Table 3).

**Table 2:** Descriptive analysis of responses of participants about the preferences towards using dental magnification devices.

Variable	*Response n (%)*				
Most preferred dental magnification device	**TTL**	**Flip up loupes**	**Corrective eyeglasses**	**DOM**	**Never use any device**
	55 (10.3)	273 (51.1)	9 (1.7)	172 (32.2)	25 (4.7)
Preferred level of magnification	**Low (3x-8x)**	**Medium (8x-16x)**		**High (16x-30x)**	**I don’t know**
	79 (14.8)	259 (48.5)		151 (28.3)	45 (8.4)
Don’t have dental magnification device to work on patient today, what will you do?	**Reschedule the patient as I don’t feel comfortable**	**Depend on the procedure, I may or may not work**		**Still work on the patient**	**Will consult my colleague**
	191 (35.8)	223 (41.8)		114 (21.3)	6 (1.1)

Note: TTL -Through The Lens; DOM—Dental Operating Microscopes.

**Table 3:** Descriptive analysis of responses of participants about the practices towards using dental magnification devices.

Variable	*Response n (%)*			
Learning of using dental magnification devices	**Through trial and error**	**During postgraduate training**	**By senior colleagues**	**By attending special continuing education courses and workshops**
	47 (8.8)	261 (48.9)	16 (3.0)	210 (39.3)
Frequency of use of dental magnification devices	**Always**	**Sometimes**	**Never**	
	247 (46.3)	240 (44.9)	47 (8.8)	

With univariate binominal regression analysis of independent variables with questions related to perception revealed that middle age group participants (35–55 years) were shown to have inadequate perception with reference to the age group 55–65 years, especially the 45–55-year age group (P<0.05; OR 0.422). Additionally, the participants in British-Isles have reported to have 2.42 times (P<0.05) higher adequate perception with reference participants in Asia sub-continent. Furthermore, participants with fellowship have 2.77 times more (P<0.01) adequate perception with reference to their counterparts with a master’s degree. Also, the specialist practicing in the public sector organizations have been found to have 5.499 times more adequate perception with reference to participants working in public sector (Table 4).

**Table 4:** Univariate logistic regression analysis of independent variables with questions related to perception about dental magnification devices.

Independent Variables		Dependent Variable		*ODDS Ratio (OR)*	*Confidence Interval (CI)*	*P Value*
		Perceptions towards using Dental Magnification Devices				
		*Inadequate n (%)*	*Adequate n (%)*			
Age	25–35 years	34 (21.0)	128 (79.0)	1.172	0.569–2.414	0.667
	35–45 years	23 (17.7)	107 (82.3)	0.802	0.363–1.771	0.586
	45–55 years	36 (27.9)	93 (72.1)	0.422	0.212–0.842	**0.014** *
	55–65 years	23 (20.4)	90 (79.6)	Ref		
Gender	Male	62 (20.5)	240 (79.5)	1.193	1.272	0.319
	Female	54 (23.3)	178 (76.7)	Ref		
Location	British Isles region	13 (14.8)	75 (85.2)	2.437	1.107–5.361	**0.027** *
	MENA Region	9 (11.5)	69 (88.5)	4.142	0.840–20.420	0.081
	Asia Sub-continent	94 (25.5)	274 (74.5)	Ref		
Level of Education	Ph.D.	28 (24.6)	86 (75.4)	0.907	0.512–1.607	0.739
	Fellowship	15 (12.9)	101 (87.1)	2.770	1.399–5.485	**0.003** **
	Board Certification	13 (13.5)	83 (86.5)	0.591	0.145–2.403	0.462
	Master Degree	60 (28.8)	148 (71.2)	Ref		
Years of Experience	More than 15 years	40 (25.2)	119 (74.8)	0.382	0.177–0.823	**0.014** *
	10 to 15 years	27 (24.3)	84 (75.7)	0.418	0.201–0.868	**0.019** *
	5 to 10 years	27 (20)	108 (80)	0.449	0.209–0.966	**0.041** *
	1–5 years	22 (17.1)	107 (82.9)	Ref		
Professional Area (Primary)	Academic only	47 (24.5)	145 (75.5)	0.721	0.426–1.221	0.224
	Clinical Practice only	25 (21)	94 (79)	1.046	0.568–1.924	0.886
	Academic and Clinical Practice	44 (19.7)	179 (80.3)	Ref		
Current Institution	Public Sector	12 (7.1)	157 (92.9)	5.499	2.794–10.822	**<0.001** ***
	Private Sector	104 (28.5)	261 (71.5)	Ref		

Note:

*P<0.05;

**P<0.01;

***P<0.001;

MENA region—Middle East and North Africa; British Isles region includes Ireland, Northern Ireland, Scotland, England and Wales.

### 3.1 Rasch analysis

**Fig 4:**
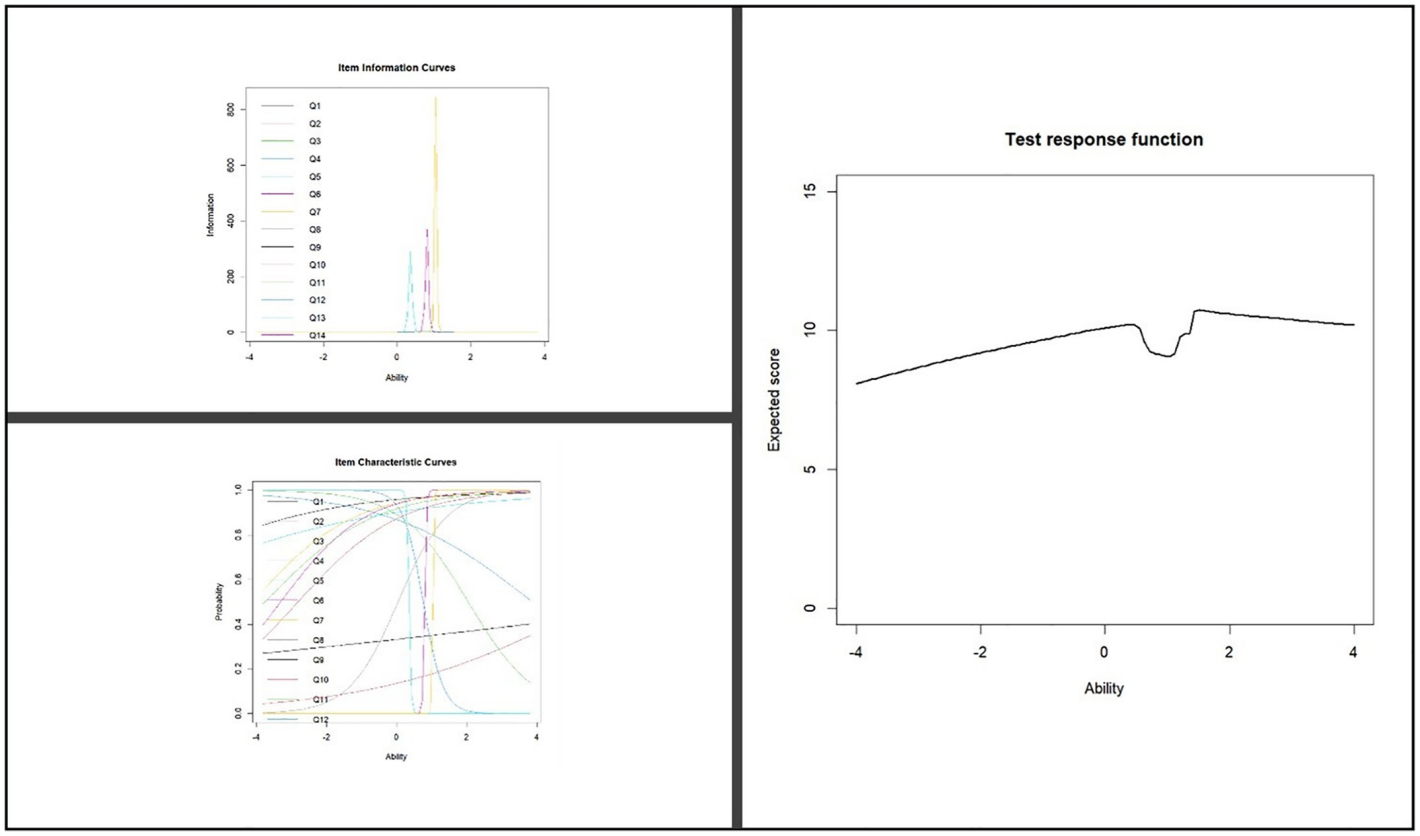
Rasch analysis.

The psychometric properties of the scale are shown in the Table 5. The difficulty parameter indicated that items Q1, Q3, Q5, Q6, and Q7 were below the recommended value of -3. This means that these questions were too easy. Also, items Q4, Q9, and Q10 had difficulty parameters above the recommended value of +3. This means that these questions were too difficult. With regards to the range of values for items for discrimination, items Q4, Q6, Q7, Q9, Q11, Q12, and Q13 were below the recommended value of 0.25. The remaining items were more than the cutoff values. However, all the items that were above the range were kept based on professional recommendation given the significance of their content in identifying the patients’ needs for endodontists and restorative procedures. According to the item goodness-of-fit test, none of the items fit the data well (P < 0.001). Across all 15 items, the items between difficulty levels -3 and +3 tapped 76.51% of the total information (Fig 4).

**Table 5:** The psychometric properties of the scale.

Items	*b*	*a*	X^2^ (df = 8)	P values
Q1	-8.22	0.38	109.01	< 0.001
Q2	-2.81	0.68	117.45	< 0.001
Q3	-3.75	0.64	98.30	< 0.001
Q4	3.87	-0.49	151.59	< 0.001
Q5	-8.16	0.27	54.08	< 0.001
Q6	-3.30	0.83	86.37	< 0.001
Q7	-4.12	0.68	130.05	< 0.001
Q8	0.04	1.44	155.52	< 0.001
Q9	8.92	0.08	158.43	< 0.001
Q10	5.71	0.32	75.90	< 0.001
Q11	2.04	-1.04	91.34	< 0.001
Q12	0.76	-3.49	86.95	< 0.001
Q13	0.35	-34.36	270.76	< 0.001
Q14	0.80	38.70	330867.39	< 0.001
Q15	1.03	58.25	75.52	< 0.001

## 4. Discussion

While there are studies in the literature that address dental professionals’ attitudes and knowledge regarding the use of various magnification tools in a particular area, they did not address the multiregional perspective [8–11]. Therefore, the present study aimed to evaluate the perception, preferences and practices of endodontists and restorative dentists in the different regions around the globe towards using dental magnification armamentarium.

Restorative and endodontic procedures require resolution well beyond the resolving power of the human eye of 0.2-1mm [15]. Devices such as DOM and loupes can enhance the resolution and hence improvise the precision required in restorative and endodontic treatment [16]. The DOM is a valuable tool because of its applications, such as magnification, better illumination, and visualization in the area of interest [2]. The high magnification helps in access preparation, locating the canals, reaching the anatomically complex areas, removing fractured instruments, and decreasing the chances of soft and hard tissue injuries [17].

In the present study, when questions regarding the perception of using dental devices were asked, the majority of the participants strongly agreed that dental magnification devices would positively influence the ergonomics (58.8%) by improving the vision of the operator (71%), prevents eye fatigue (53.6%), and decreases the operating time (63.5%). Hence, they improve the quality of work (57%) and render better prognosis (62%). By large, the participants of the present study acknowledged that the dental magnification should be considered as the standard of care in modern endodontics (60.9%). Additionally, a large number of participants felt that it should be an essential component of postgraduate training and curriculum (60.3%). These findings of the present study well align with the earlier studies of Lin et al and Castellucci, A [17, 18]. Since specialists who are using magnification daily, get accustomed to it with each passing day resulting in their dependence [19]. Due to this reason, they try to reschedule their appointment if magnification is unavailable on that day. The same was reflected in the present study where 35.8%. of the participants were in favour of rescheduling the appointment in case the magnification is not available whereas 41.8%. of the participants were of opinion that they may or may not work depending on the procedure in case they don’t have the magnification. These percentages of the responses are higher than other similar studies by Alhazzazi et al., Elkadiki N and Friedman MJ [12, 20, 21]. This could be because that our study evaluated the participants who were having at least a master degree in endodontics or restorative dentistry, whereas Alhazzazi et al. and Elkadiki N took undergraduate students in their initial training phase [12, 21]. Also, in the present study 58.8% of the participants were agreeing that use of the dental magnification devices has improved the ergonomics thus preventing dentists from developing musculoskeletal disorder. This observation is favouring the finding of an early study by Aboalshamat, K et al, suggesting that magnifications should be introduced early in dental education courses to prevent the budding dentist from musculoskeletal disorders [22].

Most of the respondents (48.6%) in the current study had learned the usage of dental magnification armamentarium during postgraduate training, whereas (39.3%) have learnt by attending continuing professional development training and courses. Similar results were obtained in a study by Alhazzazi et al. [12] wherein the postgraduate residents used magnifications (68.8%) at a higher rate and learnt by attending courses regarding the use of dental magnification armamentarium.

Regarding ergonomics, it has been observed that magnification can enhance posture and hence decrease musculoskeletal injuries [23]. Better ergonomics can help a dentist perform the treatment effectively and efficiently and work longer without muscular stains [24]. In 1999, Gary Carr introduced a DOM with Galilean optics, which had added benefits of ergonomics and configured it for dentistry. Therefore, its advantages allowed for easy use of the DOM for nearly all endodontic and restorative procedures [25]. In the present study, (58.8%) of the participants were having an improved ergonomics using the dental magnification devices. Furthermore, they were comfortable adjusting the magnification and the time needed to adjust the armamentarium. Along with this, there were no hassles for the maintenance of the magnification armamentarium. This could be because the participants had an education level of master’s and above, and they were trained in using magnification during their courses. However, few studies have reported discomfort using loupes because of their weight and non-adjustable magnification [26, 27]. Whereas, where the dental operating microscope is concerned, its high cost and need for four-handed dentistry can restrict its usage [16].

Most participants (62%) in the present study agreed that using dental magnification in complicated cases and surgical endodontics is beneficial as it improved the overall prognosis. This finding is in alignment with the results of the study by Tsesis et al. who observed a similar therapeutic outcome when using a dental operating microscope and burs to prepare retrograde cavities [26]. Similarly, with or without retrograde fillings, apicoectomies have shown better results with the dental operating microscope [28]. Studies have favoured the use of dental operating microscope compared to surgical loupes in detecting orifices and managing calcified canals to improve the prognosis [29].

In the present study, the middle-aged participants reported a significantly adequate perception for using dental magnification compared to the young age participants. This could be because the middle-aged participants have more experience and majority of them might be having postgraduate training and qualification. Similar finding was observed in a study conducted by Alrejaie et al, wherein the maximum usage of DOM was seen in the middle age group [30].

In the current study, the participants who were having more than 15 years of experience reported to have more inclination towards practicing with dental magnification compared to those who had 5 to 10 years of experience. Similar results were observed in the study done by Kersten et al. and Alrejaie et al. [27, 30]. In the present study, the participant with fellowship qualification has shown significantly positive perception towards using magnification compared to their counterparts. This could be because of the level of education. As with each increasing educational level, the specialist gets more accustomed to using specialized armamentariums to achieve better success in treatment procedures [31].

The usage of the dental operating microscope grabbed attention in 1990, and later in 2007. However, considering the MENA region, only five countries, Saudi Arabia, Lebanon, Iran, Palestine, and Jordan, have advanced specialty programs with DOM training that is not mandatory for the program’s accreditation [30]. In present study, the maximum use of magnification devices was seen in the British Isles region compared to the MENA and Indian sub-continent. This observation may be because in most of the curriculums of Indian subcontinent and partly in the MENA region, the usage of DOM has not been a part of accredited dental programs. These findings are in contrast to an earlier study showing that 90% of the endodontists in USA were using the dental operating microscope as it is a compulsory requirement for the accredited endodontic training program [28].

Furthermore, in the current study the magnification device usage was significantly more in the public sector as compared to the private sector. Probably this observation could be because the government sectors are generally fully equipped with armamentariums. A similar finding has been reported wherein the knowledge, attitude and practice were assessed for the use of DOM in endodontic procedure for children [32].

### 4.1 Study strengths

One of the key strengths of our study is its global, multicenter approach, which allows us to capture a wide range of perspectives from endodontists and restorative dentists across different regions. This diversity in sampling is crucial as it provides a more generalizable understanding of how dental magnification tools are perceived and utilized worldwide, rather than limiting the findings to a specific region or demographic. Moreover, our study focused on professionals with advanced degrees (master’s and above), ensuring that the participants had substantial clinical experience and knowledge. This focus on well-trained practitioners increases the reliability of our findings, as their responses are informed by years of specialized practice rather than theoretical knowledge or limited experience. Additionally, by incorporating participants from both public and private sectors, we could compare and contrast the accessibility and usage of dental magnification tools in different practice settings, adding depth to our analysis.

### 4.2 Clinical implications

The results of our study have significant implications for dental education, clinical practice, and policy-making. The strong endorsement of dental magnification devices as a standard of care by a majority of participants suggests that these tools are not only beneficial but potentially essential for modern dental practice. As a result, there is a clear need for dental education programs to integrate the use of these devices into their curricula, particularly at the postgraduate level. This integration will ensure that future dentists are well-equipped with the necessary skills to utilize magnification tools effectively, ultimately improving patient outcomes. Furthermore, our findings indicate a potential gap in access to magnification tools, particularly in certain regions and private practice settings. Addressing these disparities through policy changes, such as mandating the availability of these devices in all accredited dental programs and encouraging their use in private practices, could lead to more consistent and widespread adoption of these technologies. Finally, our study highlights the importance of ongoing professional development and training in the use of dental magnification devices. As demonstrated by the participants who acquired their skills through continuing education, lifelong learning is crucial for staying abreast of technological advancements in dentistry.

### 4.3 Limitations and future directions

Although it is an online survey and thus adopted the snowball technique for data collection, it still managed to have a ’representative samples from all the regions considered in the study. Being an online survey, response bias could be a possibility which can influence the results. Therefore, such multicentre study with stratified random sampling is desirable and recommended to generalize the results for a wider population.

## 5. Conclusions

The perspective of the majority of participants in the current study was that dental magnification devices improve vision, ergonomics, quality of work, prevent eye fatigue, decrease operating time, improve prognosis, and thus should be considered the standard of care in modern endodontic and restorative dentistry practice. Therefore, it is recommended that the use of dental magnification devices be regarded as a requisite component of accredited postgraduate dental training programs and curricula.

## Supporting information

S1 Data Raw data in excel sheet. (XLSX)

S1 Questionnaire (DOCX)
