# Supplementary material for: Practices and preferences in the use of magnification among endodontists and restorative dentists: A multicentre study
Source: PLoS One. 2025 Jan 7;20(1):e0311391. doi: 10.1371/journal.pone.0311391 (PMC11706448; doi:10.1371/journal.pone.0311391)
Supplement: S1 Questionnaire — (DOCX) [file pone.0311391.s002.docx]

**Perception, preferences and practices of Endodontists and Restorative Dentists towards the use of dental magnification armamentarium- A Multicenter Study**

**Section A: Demographic Data**

| 1. Age  25-35 years  35-45 years  45-55 years  Above 55 years  2. Gender  Male  Female  3. Location  British Isles region  MENA region  Indian subcontinent  4. Education  Ph.D.  Fellowship  Board certification  Master degree  5. Years of Experience  More than 15 years  10-15 years  5-10 years  1-5 years  6. Professional Area (Primary)  Academic only  Clinical practice only  Academic & Clinical Practice  7. Type of Organization  Public sector  Private sector |
| --- |
|  |
|  |
|  |
|  |
|  |
|  |
|  |
|  |
|  |
|  |
|  |
|  |
|  |
|  |
|  |
|  |
|  |
|  |
|  |
|  |
|  |
|  |
|  |
|  |
|  |
|  |
|  |
|  |
|  |
|  |

**Section B: Perceptions of Endodontists and Restorative Dentists towards using dental magnification**

|  |  |
| --- | --- |
|  |  |

1. Dental magnification devices will improve the vision of operator

- Strongly agree
- Agree
- Neutral
- Disagree
- Strongly disagree

1. Dental magnification devices will prevent eye fatigue / strain of operator

- Strongly agree
- Agree
- Neutral
- Disagree
- Strongly disagree

1. Dental magnification devices will improve the ergonomics

- Strongly agree
- Agree
- Neutral
- Disagree
- Strongly disagree

1. Dental magnification will enhance the quality of work

- Strongly agree
- Agree
- Neutral
- Disagree
- Strongly disagree

1. Dental magnification devices will make the treatment of complicated cases easy

- Strongly agree
- Agree
- Neutral
- Disagree
- Strongly disagree

1. Dental magnification devices will result in the better prognosis of endodontic retreatment and surgical endodontic cases

- Strongly agree
- Agree
- Neutral
- Disagree
- Strongly disagree

1. Dental magnification devices are considered standard of care in modern Endodontics and Restorative dentistry practice

- Strongly agree
- Agree
- Neutral
- Disagree
- Strongly disagree

1. Takes too much time to position the patient and Specialist for the dental magnification devices

- Strongly agree
- Agree
- Neutral
- Disagree
- Strongly disagree

1. Difficult to position the dental magnification devices during procedures

- Strongly agree
- Agree
- Neutral
- Disagree
- Strongly disagree

1. Maintenance of dental magnification devices is difficult

- Strongly agree
- Agree
- Neutral
- Disagree
- Strongly disagree

11. Dental magnification devices will decrease the operator time

- Strongly agree
- Agree
- Neutral
- Disagree
- Strongly disagree

1. It Should be an essential component of Postgraduate training and curriculum in Endodontic and Restorative Dentistry

- Strongly agree
- Agree
- Neutral
- Disagree
- Strongly disagree

1. CPD training courses and workshops needed

- Strongly agree
- Agree
- Neutral
- Disagree
- Strongly disagree

1. Special training needed for the dental assistants for the maintenance of dental magnification devices.

- Strongly agree
- Agree
- Neutral
- Disagree
- Strongly disagree

1. Cost-effective to use the dental magnification devices.

- Strongly agree
- Agree
- Neutral
- Disagree
- Strongly disagree

**Section C: Preferences of Endodontists and Restorative Dentists towards using dental magnification**

1. Area of clinical interest

- Operative/ Restorative Procedure
- Non-surgical endodontic treatment and retreatment
- Surgical endodontics

1. Most preferred dental magnification device

- Flip up loupes
- TTL
- DOM
- Corrective eyeglasses
- Never use any device

1. Preferred level of magnification

- Low (3x-8x)
- Medium (8x-16 x)
- High (16 x- 30 x)
- Don’t work with magnification

1. Don’t have dental magnification device to work on patient today, what will you do?

- Reschedule the patient as I don’t feel comfortable
- Depend on the procedure, I may or may not work
- Still work on the patient
- Will consult my colleague

**Section D: Practices of Endodontists and Restorative Dentists towards using dental magnification**

1. Learning of using dental magnification devices

- Through trial and error
- During post graduate training
- By senior colleagues
- By attending special continuing education courses and workshops

1. Frequency of use of dental magnification devices

- For all Endodontic procedures
- For Endodontic procedures on posterior teeth only
- For Endodontic procedures on anterior teeth only
- Never

3. Procedures for which the dental magnification devices are used

- Locating hidden canals
- Negotiating calcified canals
- Retrieval of broken instruments in the canal
- By passing ledges and perforation repair
- For canal preparation and obturation
- For surgical endodontic
- For crack detection
- For caries removal
- For tooth preparation for coronal restorations and veneers
