# Supplementary material for: Practices and preferences in the use of magnification among endodontists and restorative dentists: A multicentre study
Source: PLoS One. 2025 Jan 7;20(1):e0311391. doi: 10.1371/journal.pone.0311391 (PMC11706448; doi:10.1371/journal.pone.0311391)
Supplement: Attachment — Submitted filename: Comments to Author (PONE-D-23-42581 ) .docx (10 Mart 2024).pdf [file pone.0311391.s003.pdf]

Manuscript Number: PONE-D-23-42581

Article Type: Research Article

Manuscript Title: Perception, preferences and practices of Endodontists and Restorative Dentists towards the use of dental magnification armamentarium- A multicentre study

Comments to Author

Abstract

The sentence in the conclusion may only be true for the DOM.

Introductıon

“canters” should be rearranged for english (line 75)

Materials and Methods

This study was well designed in line with these objectives of the study. However, instead of "Who uses magnification more?", "How does magnification affect the quality and success rate of treatment?" question may contribute more to the literature.

Results

Rash analysis as well as the whole organization and presentation of the results were apropriate.

Discussion

In general, this section does not provide a discussion or comment on how the results of this study contribute to the literature. Also, most of the references are misused. Sentences do not match the number of references.

For example; Reference 16 is used incorrectly in the first sentence of the first paragraph of the discussion section (This reference belongs to the Rash analysis).

In the second paragraph, it seems that all the references are used incorrectly. References 20,21 in the references section are by Lins and Castellucci. But the situation is different in the discussion part” This follows the study by Farook et al. and Alhazzazi et al [20,21]” (lines 215,216).

In the last sentence of the second paragraph, the authors gave only 1 reference for “ few studies”. There must be more than 1 reference (Line 223). Moreover, 37 references were used in the article. However, 32 references were reported in the references section.

Finally, the discussion section was written carelessly. There is no explanation or assesment regarding the contribution of this study to the literature.

Regards.
