# Supplementary material for: Practices and preferences in the use of magnification among endodontists and restorative dentists: A multicentre study
Source: PLoS One. 2025 Jan 7;20(1):e0311391. doi: 10.1371/journal.pone.0311391 (PMC11706448; doi:10.1371/journal.pone.0311391)
Supplement: Attachment — Submitted filename: Reply to reviewers.docx [file pone.0311391.s004.docx]

Dear Prof,

Thanks for sparing your valuable time in reviewing our manuscript titled ***“Perception, preferences and practices of Endodontists and Restorative Dentists towards the use of dental magnification armamentarium- A multicentre study" (Submission ID PONE-D-23-42581)***. We would like to express our sincere thanks to the editor and the reviewers for the constructive and positive comments. Please find point-to-point clarification/reply for the concerns raised. All modifications made in the manuscript are highlighted in yellow colour.

Thanks & Regards

| **S. NO** | **Reviewer number** | **Reviewer comments** | **Authors response** | **Changes made with Page and Line No.** |
| --- | --- | --- | --- | --- |
| 1. | **Journal Requirements** | Please ensure that your manuscript meets PLOS ONE's style requirements, including those for file naming. The PLOS ONE style templates can be found at  <https://journals.plos.org/plosone/s/file?id=wjVg/PLOSOne_formatting_sample_main_body.pdf> and  <https://journals.plos.org/plosone/s/file?id=ba62/PLOSOne_formatting_sample_title_authors_affiliations.pdf> | Thanks for the comment. The manuscript has been revised as per the journal policy. | Entire manuscript |
| 2. | **Journal Requirements** | Please provide additional details regarding participant consent. In the ethics statement in the Methods and online submission information, please ensure that you have specified what type of consent you obtained (for instance, written or verbal, and if verbal, how it was documented and witnessed). | Thanks for the comment. The detailed description about the consent has been added in the methodology – section No. 2.4.  “After opening the survey link, the participant read the consent statement. Only if he/she agreed to the consent, participant got the access to the questionnaire. In otherwise situation where the potential participant had chosen to decline the consent, the survey got terminated.” | Page 5, Line 125-127 |
| 3. | **Journal Requirements** | We note that the grant information you provided in the ‘Funding Information’ and ‘Financial Disclosure’ sections do not match.  When you resubmit, please ensure that you provide the correct grant numbers for the awards you received for your study in the ‘Funding Information’ section. | Thanks for the comment. With regard to the “funding” I would to like mention that the university had initially proposed a special funding category “Fast-track Research Funding Program” and provisionally agreed to fund this particular research project after successful publication. Keeping that in view, we mentioned the funding statement.  However, due to recent changes / developments in the higher administration, that particular funding category has been postposed till further notice.  So, considering the recent development at my university, I will prefer to retract this funding statement and will suggest to mention as “self-funded”.  I hope the editorial board will understand the situation and will give a consent to modify the “funding statement”. | Page 10, Line 297 |
| 4. | **Journal Requirements** | Thank you for stating the following financial disclosure:  "This work was funded by the Deanship of Scientific Research at Jouf University through the Fast-track Research Funding Program"  Please state what role the funders took in the study. If the funders had no role, please state: "The funders had no role in study design, data collection and analysis, decision to publish, or preparation of the manuscript."  If this statement is not correct you must amend it as needed.  Please include this amended Role of Funder statement in your cover letter; we will change the online submission form on your behalf. | Thanks for the comment. As described in the previous comment, I have modified that funding statement as “self-funded”. | Page 10, Line 297 |
| 5. | **Journal Requirements** | In the online submission form, you indicated that "The data set used in the current study will be made available at the reasonable request."  All PLOS journals now require all data underlying the findings described in their manuscript to be freely available to other researchers, either 1. In a public repository, 2. Within the manuscript itself, or 3. Uploaded as supplementary information.  This policy applies to all data except where public deposition would breach compliance with the protocol approved by your research ethics board. If your data cannot be made publicly available for ethical or legal reasons (e.g., public availability would compromise patient privacy), please explain your reasons on resubmission and your exemption request will be escalated for approval. | Thanks for the comment. As suggested, the raw data file (excel sheet) has been provided as the “supplementary information”. | Page 10, Uploaded as supplementary information |
| 6. | **Journal Requirements** | Your ethics statement should only appear in the Methods section of your manuscript. If your ethics statement is written in any section besides the Methods, please move it to the Methods section and delete it from any other section. Please ensure that your ethics statement is included in your manuscript, as the ethics statement entered into the online submission form will not be published alongside your manuscript. | Thanks for the concern. The initial submitted file had the duplication of the ethics information – methodology and declarations. As suggested, the ethics statement has been removed from the declaration section. | Page 10 |
| 7. | **Journal Requirements** | Please include captions for your Supporting Information files at the end of your manuscript, and update any in-text citations to match accordingly. Please see our Supporting Information guidelines for more information: <http://journals.plos.org/plosone/s/supporting-information>. | Thanks for the comment. As suggested, the supplementary file has been named. | Page 10 |

Reviewer Comments

| **S. NO** | **Reviewer number** | **Reviewer comments** | **Authors response** | **Changes made with Page and Line No.** |
| --- | --- | --- | --- | --- |
| 1. | **1** | The manuscript requires thorough revision by an English language expert to ensure clarity, coherence, and grammatical accuracy. | Thanks for the comment. As suggested, the manuscript has been extensively edited by a native English speaker. | Entire Manuscript. |
| 2. | **1** | **Abstract section:**  1. Material and methods: A multicenter cross-sectional study  2. Remove "at King Abdulaziz University, Jeddah"  3. This sentence ("Following the ethical approval of the present study by  the local scientific research ethics committee") can be removed as it was already mentioned earlier  4. Specify the centres involved in the study  5. Rewrite the conclusion section | Thanks for the comment. As suggested, the corrections in the methodology and conclusion have been done. The conclusion has been re-written in Abstract as well as at the end conclusion. | Page 2, Line 46,47,48 |
| 3. | **1** | **Introduction section:**  “Although few studies in the scientific literature have shown predictable results when the dental magnification has been incorporated and integrated into the undergraduate, postgraduate dental curriculum and clinical scenarios, but still the evidence based scientific literature supporting the effect of dental magnification on the performance of dentists is very weak.”  This sentence is inaccurate, and furthermore, the paper referenced does not substantiate its claim; | Thanks for the comment.  The sentence has been re-written, and the reference has also been corrected | Page 3, Line 72,73 |
| 4. | **1** | **MM section:**  “Out of 15 questions, only three questions namely no. 8 -10 were negatively directed.” What did the authors mean with “negatively directed”?  "Each question had a correct answer, so while calculating the overall score of each sample, “1” score is given for the “correct” response while “0” score was given for the “incorrect” response."  Data management of this study is quite questionable. How does it work? | Thanks for the comment. With regard to the concern raised by the respected reviewer, I would like to clarify the terminology “negatively directed”.  In order to eliminate the “response set bias” the negatively worded questions were introduced.  The section of data management is modified to bring clarity.  “The response to all questions regarding the perception about dental magnification were gathered on a 5-point Likert scale; However, each question had a correct response. Accordingly, prior to data analysis, the responses were analysed and were marked as “correct” or “incorrect”. For the sake of data analysis, the responses were assigned with a dummy code of “1” for correct and “0” for an incorrect response. Overall score for each respondent was calculated by adding the score attained by each question. Eventually, the overall scores were categorized into 2 scales namely “adequate” and “inadequate” using the equation mentioned below”.  The method adopted has been reproduced from published research [10.3390/ijerph17207415](http://dx.doi.org/10.3390/ijerph17207415) |  |
| 5. | **1** | **Results section:**  This section appears to be excessively long and overly detailed, as it describes all the contents of the Tables and Figures, which may not be necessary.  "Around 53.6 to 71% of the participants strongly agreed that dental magnification devices will improve the vision, ergonomics, quality of work, prevent eye fatigue, better prognosis, standard of care in modern endodontic, decreases the operating time, and essential component of postgraduate training and curriculum." How is this (53.6 to 71%) possible? | Thanks for the comment. As suggested, the section has been modified. |  |
| 6. | **1** | **Discussion section**  It is essential to discuss the results in relation to the existing literature that has been previously published. | Thanks for the comment. Results have been discussed in relation to published existing literature |  |
| 7. | **2** | **Abstract**  The sentence in the conclusion may only be true for the DOM. | Thank you for the comment. The sentence in the conclusion part of the abstract has been corrected and it has been rewritten at the end in conclusion. | Page 2, Line 46,47,48  Page 9, Lines 282-285 |
| 8. | **2** | **Introduction**  “canters” should be rearranged for english (line 75) | Thanks for the comment. As suggested, the typographical error has been corrected. | Page 3, Line 75 |
| 9. | **2** | **Materials and Methods**  This study was well designed in line with these objectives of the study. However, instead of "Who uses magnification more?", "How does magnification affect the quality and success rate of treatment?" question may contribute more to the literature. | Thanks for the comment. With all due respect to the concern raised by the reviewer, I would like to mention that we have included several questions in the “Perception” segment of the questionnaire especially PQ No. 1, 2, 3, 4, and 11, that potentially contribute to the success of the treatment by using dental magnification. Kindly refer that table 1 for the complete set of questions and their responses from the participants. | Page 14, Table 1 |
| 10. | **2** | **Results**  Rash analysis as well as the whole organization and presentation of the results were appropriate. | We thank respected reviewer for encouraging comments. |  |
| 11. | **2** | **Discussion**  In general, this section does not provide a discussion or comment on how the results of this study contribute to the literature. Also, most of the references are misused. Sentences do not match the number of references. For example;  Reference 16 is used incorrectly in the first sentence of the first paragraph of the discussion section (This reference belongs to the Rash analysis).  In the second paragraph, it seems that all the references are used incorrectly. References 20,21 in the references section are by Lins and Castellucci. But the situation is different in the discussion part” This follows the study by Farook et al. and Alhazzazi et al [20,21]” (lines 215,216).  In the last sentence of the second paragraph, the authors gave only 1 reference for “few studies”. There must be more than 1 reference (Line 223). Moreover, 37 references were used in the article. However, 32 references were reported in the references section.  Finally, the discussion section was written carelessly. There is no explanation or assessment regarding the contribution of this study to the literature. | Thank you for the comments.  The numbering of the references has been corrected according to the sentences.  For example  Reference 16 has been corrected.  References 20,21 have been corrected.  Reference 20 =19  Reference 21=20  Thank you for the comment. The numbers of the references have been corrected.  Thank you for the comment.  The discussion section has been rewritten carefully matching the sentences with the number of references. Also mentioned that this study will give a multiregional regional perspective of endodontists and restorative dentist about dental magnification in the literature. | Page 7, line 210,211  Page 8, line 221,222  Page 10,11,12  Line 323-325  Line 347-349  Line 361-362  Line 368-375  Line 376-380  Line 392-405  Page 7,8,9,10  Line 210-217  Line 225-229  Line232-235  Line 239-241  Line 244-246  Line 247-249  Line 251-254  Line 260-264  Line 263-269  Line 278-283  Line 296-299 |
