# Supplementary material for: Practices and preferences in the use of magnification among endodontists and restorative dentists: A multicentre study
Source: PLoS One. 2025 Jan 7;20(1):e0311391. doi: 10.1371/journal.pone.0311391 (PMC11706448; doi:10.1371/journal.pone.0311391)
Supplement: Attachment — Submitted filename: Response to Comments.docx [file pone.0311391.s005.docx]

Comments:

**Reviewer #3:**

**Comment 1:** The authors have adequately responded to the comments however the discussion requires more information. The authors should describe the strengths of their study as well as the implications of the results presented. That section can be made stronger and more impactful.

**Authors Response:** We appreciate the reviewer's insightful feedback and agree that the Discussion section could be further strengthened. We have revised the section to include a more comprehensive description of the study's strengths and the broader implications of our findings.

**Study Strengths:** One of the key strengths of our study is its global, multicenter approach, which allows us to capture a wide range of perspectives from endodontists and restorative dentists across different regions. This diversity in sampling is crucial as it provides a more generalizable understanding of how dental magnification tools are perceived and utilized worldwide, rather than limiting the findings to a specific region or demographic. Moreover, our study focused on professionals with advanced degrees (master's and above), ensuring that the participants had substantial clinical experience and knowledge. This focus on well-trained practitioners increases the reliability of our findings, as their responses are informed by years of specialized practice rather than theoretical knowledge or limited experience. Additionally, by incorporating participants from both public and private sectors, we could compare and contrast the accessibility and usage of dental magnification tools in different practice settings, adding depth to our analysis.

**Implications of the Results:** The results of our study have significant implications for dental education, clinical practice, and policy-making. The strong endorsement of dental magnification devices as a standard of care by a majority of participants suggests that these tools are not only beneficial but potentially essential for modern dental practice. As a result, there is a clear need for dental education programs to integrate the use of these devices into their curricula, particularly at the postgraduate level. This integration will ensure that future dentists are well-equipped with the necessary skills to utilize magnification tools effectively, ultimately improving patient outcomes. Furthermore, our findings indicate a potential gap in access to magnification tools, particularly in certain regions and private practice settings. Addressing these disparities through policy changes, such as mandating the availability of these devices in all accredited dental programs and encouraging their use in private practices, could lead to more consistent and widespread adoption of these technologies. Finally, our study highlights the importance of ongoing professional development and training in the use of dental magnification devices. As demonstrated by the participants who acquired their skills through continuing education, lifelong learning is crucial for staying abreast of technological advancements in dentistry.

We believe that these additions provide a more robust and impactful discussion of our study.

**Reviewer #4:**

The authors are commended for a well written manuscript and the study will be of good interest to the readers.

Following the corrections made by the authors addressing most of the comments.

**Comment 1:** I will recommend considering the modification of the title to: Practices and preferences in the use of magnification among endodontists and restorative dentists - A multicentre study.

**Authors Response:** We appreciate the positive feedback on our manuscript and thank you for the thoughtful suggestion regarding the title. We agree that the proposed title is more concise and focused, effectively capturing the essence of the study. Therefore, we have revised the title as follows:

Revised Title: "Practices and Preferences in the Use of Magnification Among Endodontists and Restorative Dentists: A Multicentre Study"

This modification aligns with the manuscript's content and enhances the clarity and impact of the title. Thank you for your valuable input.

**Comment 2:** In addition, there is an abbreviation "it's" in line 276 that should be corrected.

**Authors Response:** Thank you for bringing this to our attention. We have corrected the abbreviation "it’s" in line 300 to the appropriate form "it is" The sentence now accurately reflects the intended meaning without any grammatical errors. We appreciate your careful review and attention to detail.

**Comment 3:** A section that can be further improved is the conclusion, authors are advised to avoid the use of authoritative words such as "must". The last sentence can be rephrased in a way that subtle words like "requisite, obliged, required"

**Authors Response:** We appreciate the suggestion to refine the language in the conclusion to make it more balanced and less authoritative. We have revised the last sentence to incorporate more subtle wording, ensuring that the recommendation is still clear while being less directive.

**Revised Conclusion:** The perspective of the majority of participants in the current study was that dental magnification devices improve vision, ergonomics, quality of work, prevent eye fatigue, decrease operating time, improve prognosis, and thus should be considered the standard of care in modern endodontic and restorative dentistry practice. Therefore, it is recommended that the use of dental magnification devices be regarded as a requisite component of accredited postgraduate dental training programs and curricula.

This revision aligns with your feedback, creating a more measured and professional tone in our conclusion. Thank you for your valuable input.
