# Supplementary material for: Practices and preferences in the use of magnification among endodontists and restorative dentists: A multicentre study
Source: PLoS One. 2025 Jan 7;20(1):e0311391. doi: 10.1371/journal.pone.0311391 (PMC11706448; doi:10.1371/journal.pone.0311391)
Supplement: Peer review history

# Peer review: Decision Letter 0

**DOI:** 10.1371/journal.pone.0311391.r001

**Authors:** Tanay Chaubal

10 Mar 2024

PONE-D-23-42581Perception, preferences and practices of Endodontists and Restorative Dentists towards the use of dental magnification armamentarium- A multicentre studyPLOS ONE

Dear Dr. Iqbal,

Thank you for submitting your manuscript to PLOS ONE. After careful consideration, we feel that it has merit but does not fully meet PLOS ONE’s publication criteria as it currently stands. Therefore, we invite you to submit a revised version of the manuscript that addresses the points raised during the review process.

Please submit your revised manuscript by Apr 24 2024 11:59PM. If you will need more time than this to complete your revisions, please reply to this message or contact the journal office at plosone@plos.org. When you're ready to submit your revision, log on to https://www.editorialmanager.com/pone/ and select the 'Submissions Needing Revision' folder to locate your manuscript file.

Please include the following items when submitting your revised manuscript:

- A rebuttal letter that responds to each point raised by the academic editor and reviewer(s). You should upload this letter as a separate file labeled 'Response to Reviewers'.
- A marked-up copy of your manuscript that highlights changes made to the original version. You should upload this as a separate file labeled 'Revised Manuscript with Track Changes'.
- An unmarked version of your revised paper without tracked changes. You should upload this as a separate file labeled 'Manuscript'.

If you would like to make changes to your financial disclosure, please include your updated statement in your cover letter. Guidelines for resubmitting your figure files are available below the reviewer comments at the end of this letter.

If applicable, we recommend that you deposit your laboratory protocols in protocols.io to enhance the reproducibility of your results. Protocols.io assigns your protocol its own identifier (DOI) so that it can be cited independently in the future. For instructions see: https://journals.plos.org/plosone/s/submission-guidelines#loc-laboratory-protocols. Additionally, PLOS ONE offers an option for publishing peer-reviewed Lab Protocol articles, which describe protocols hosted on protocols.io. Read more information on sharing protocols at https://plos.org/protocols?utm_medium=editorial-email&utm_source=authorletters&utm_campaign=protocols.

We look forward to receiving your revised manuscript.

Kind regards,

Tanay Chaubal

Academic Editor

PLOS ONE

Journal Requirements:

When submitting your revision, we need you to address these additional requirements.

1. Please ensure that your manuscript meets PLOS ONE's style requirements, including those for file naming. The PLOS ONE style templates can be found at 

https://journals.plos.org/plosone/s/file?id=wjVg/PLOSOne_formatting_sample_main_body.pdf and 

https://journals.plos.org/plosone/s/file?id=ba62/PLOSOne_formatting_sample_title_authors_affiliations.pdf

2. Please provide additional details regarding participant consent. In the ethics statement in the Methods and online submission information, please ensure that you have specified what type of consent you obtained (for instance, written or verbal, and if verbal, how it was documented and witnessed).

3. We note that the grant information you provided in the ‘Funding Information’ and ‘Financial Disclosure’ sections do not match. 

When you resubmit, please ensure that you provide the correct grant numbers for the awards you received for your study in the ‘Funding Information’ section.

4. Thank you for stating the following financial disclosure: 

"This work was funded by the Deanship of Scientific Research at Jouf University through the Fast-track Research  Funding Program"

Please state what role the funders took in the study.  If the funders had no role, please state: ""The funders had no role in study design, data collection and analysis, decision to publish, or preparation of the manuscript."" 

If this statement is not correct you must amend it as needed. 

Please include this amended Role of Funder statement in your cover letter; we will change the online submission form on your behalf.

5. In the online submission form, you indicated that "The data set used in the current study will be made available at the reasonable request."

. 

All PLOS journals now require all data underlying the findings described in their manuscript to be freely available to other researchers, either 1. In a public repository, 2. Within the manuscript itself, or 3. Uploaded as supplementary information.

This policy applies to all data except where public deposition would breach compliance with the protocol approved by your research ethics board. If your data cannot be made publicly available for ethical or legal reasons (e.g., public availability would compromise patient privacy), please explain your reasons on resubmission and your exemption request will be escalated for approval.

6. Your ethics statement should only appear in the Methods section of your manuscript. If your ethics statement is written in any section besides the Methods, please move it to the Methods section and delete it from any other section. Please ensure that your ethics statement is included in your manuscript, as the ethics statement entered into the online submission form will not be published alongside your manuscript.

7. Please include captions for your Supporting Information files at the end of your manuscript, and update any in-text citations to match accordingly. Please see our Supporting Information guidelines for more information: http://journals.plos.org/plosone/s/supporting-information.

**Additional Editor Comments:**

==============================

ACADEMIC EDITOR: I thank you for your submitting your valuable research to the journal. However, there are queries raised by the reviewers' which if addressed would make your manuscript more robust. 

==============================

[Note: HTML markup is below. Please do not edit.]

Reviewers' comments:

Reviewer's Responses to Questions

**Comments to the Author**

1. Is the manuscript technically sound, and do the data support the conclusions?

The manuscript must describe a technically sound piece of scientific research with data that supports the conclusions. Experiments must have been conducted rigorously, with appropriate controls, replication, and sample sizes. The conclusions must be drawn appropriately based on the data presented. 

Reviewer #1: Partly

Reviewer #2: Partly

**********

2. Has the statistical analysis been performed appropriately and rigorously? 

Reviewer #1: I Don't Know

Reviewer #2: I Don't Know

**********

3. Have the authors made all data underlying the findings in their manuscript fully available?

The PLOS Data policy requires authors to make all data underlying the findings described in their manuscript fully available without restriction, with rare exception (please refer to the Data Availability Statement in the manuscript PDF file). The data should be provided as part of the manuscript or its supporting information, or deposited to a public repository. For example, in addition to summary statistics, the data points behind means, medians and variance measures should be available. If there are restrictions on publicly sharing data—e.g. participant privacy or use of data from a third party—those must be specified.

Reviewer #1: Yes

Reviewer #2: Yes

**********

4. Is the manuscript presented in an intelligible fashion and written in standard English?

PLOS ONE does not copyedit accepted manuscripts, so the language in submitted articles must be clear, correct, and unambiguous. Any typographical or grammatical errors should be corrected at revision, so please note any specific errors here.

Reviewer #1: No

Reviewer #2: No

**********

5. Review Comments to the Author

Please use the space provided to explain your answers to the questions above. You may also include additional comments for the author, including concerns about dual publication, research ethics, or publication ethics. (Please upload your review as an attachment if it exceeds 20,000 characters)

Reviewer #1: Thank you for submitting the paper entitled “Perception, preferences and practices of Endodontists and Restorative Dentists towards the use of dental magnification armamentarium - A multicentre study” to Plos One. In this paper, the authors aimed to assess how endodontists and restorative dentists perceive, prefer, and use different dental magnification instruments in different locations around the world (MENA - Middle East and Northern Africa, British-Isles, and Indian sub-continent).

While acknowledging the intricacies involved in experiment planning, the significant number of participants, and the effort invested in manuscript preparation, the current study unfortunately exhibits drawbacks that hinder my recommendation for its publication.

Given the existence of several previously published papers on this topic, the relevance of the data obtained in this study is diminished, particularly for a high-impact-factor journal such as PLOS ONE. Here are some points that need to be addressed:

- The manuscript requires thorough revision by an English language expert to ensure clarity, coherence, and grammatical accuracy;

- Abstract section:

1.Material and methods: A multicenter cross-sectional study

2. Remove "at King Abdulaziz University, Jeddah"

3. This sentence ("Following the ethical approval of the present study by the local scientific research ethics committee") can be removed as it was already mentioned earlier

4. Specify the centers involved in the study

5. Rewrite the conclusion section

- Introduction section:

“Although few studies in the scientific literature have shown predictable results when the dental magnification has been incorporated and integrated into the undergraduate, postgraduate dental curriculum and clinical scenarios, but still the evidence based scientific literature supporting the effect of dental magnification on the performance of dentists is very weak.”

This sentence is inaccurate, and furthermore, the paper referenced does not substantiate its claim;

- MM section:

“Out of 15 questions, only three questions namely no. 8 -10 were negatively directed.”

What did the authors mean with “negatively directed”?

"Each question had a correct answer, so while calculating the overall score of each sample, “1” score is given for the “correct” response while “0” score was given for the “incorrect” response."

Data management of this study is quite questionable. How does it work?

- Results section:

This section appears to be excessively long and overly detailed, as it describes all the contents of the Tables and Figures, which may not be necessary.

"Around 53.6 to 71% of the participants strongly agreed that dental magnification devices will improve the vision, ergonomics, quality of work, prevent eye fatigue, better prognosis, standard of care in modern endodontic, decreases the operating time, and essential component of postgraduate training and curriculum."

How is this (53.6 to 71%) possible?

- Discussion section

It is essential to discuss the results in relation to the existing literature that has been previously published.

Reviewer #2: Manuscript Number: PONE-D-23-42581

Article Type: Research Article

Manuscript Title: Perception, preferences and practices of Endodontists and Restorative Dentists towards the use of dental magnification armamentarium- A multicentre study

Comments to Author

Abstract

The sentence in the conclusion may only be true for the DOM.

Introductıon

“canters” should be rearranged for english (line 75)

Materials and Methods

This study was well designed in line with these objectives of the study. However, instead of "Who uses magnification more?", "How does magnification affect the quality and success rate of treatment?" question may contribute more to the literature.

Results

Rash analysis as well as the whole organization and presentation of the results were apropriate.

Discussion

In general, this section does not provide a discussion or comment on how the results of this study contribute to the literature. Also, most of the references are misused. Sentences do not match the number of references.

For example; Reference 16 is used incorrectly in the first sentence of the first paragraph of the discussion section (This reference belongs to the Rash analysis).

In the second paragraph, it seems that all the references are used incorrectly. References 20,21 in the references section are by Lins and Castellucci. But the situation is different in the discussion part” This follows the study by Farook et al. and Alhazzazi et al [20,21]” (lines 215,216).

In the last sentence of the second paragraph, the authors gave only 1 reference for “ few studies”. There must be more than 1 reference (Line 223). Moreover, 37 references were used in the article. However, 32 references were reported in the references section.

Finally, the discussion section was written carelessly. There is no explanation or assesment regarding the contribution of this study to the literature.

Regards.

**********

6. PLOS authors have the option to publish the peer review history of their article (what does this mean?). If published, this will include your full peer review and any attached files.

If you choose “no”, your identity will remain anonymous but your review may still be made public.

**Do you want your identity to be public for this peer review?** For information about this choice, including consent withdrawal, please see our Privacy Policy.

Reviewer #1: No

Reviewer #2: **Yes: **Hatice Dogan Buzoglu DDS, PhD

**********

[NOTE: If reviewer comments were submitted as an attachment file, they will be attached to this email and accessible via the submission site. Please log into your account, locate the manuscript record, and check for the action link "View Attachments". If this link does not appear, there are no attachment files.]

While revising your submission, please upload your figure files to the Preflight Analysis and Conversion Engine (PACE) digital diagnostic tool, https://pacev2.apexcovantage.com/. PACE helps ensure that figures meet PLOS requirements. To use PACE, you must first register as a user. Registration is free. Then, login and navigate to the UPLOAD tab, where you will find detailed instructions on how to use the tool. If you encounter any issues or have any questions when using PACE, please email PLOS at figures@plos.org. Please note that Supporting Information files do not need this step.

Attachment Submitted filename: Comments to Author (PONE-D-23-42581 ) .docx (10 Mart 2024).pdf

# Author comment: Author response to Decision Letter 0

**DOI:** 10.1371/journal.pone.0311391.r002

2 May 2024

Dear Prof,

Thanks for sparing your valuable time in reviewing our manuscript titled “Perception, preferences and practices of Endodontists and Restorative Dentists towards the use of dental magnification armamentarium- A multicentre study" (Submission ID PONE-D-23-42581). We would like to express our sincere thanks to the editor and the reviewers for the constructive and positive comments. Please find point-to-point clarification/reply for the concerns raised. All modifications made in the manuscript are highlighted in yellow colour.

Thanks & Regards

Attachment Submitted filename: Reply to reviewers.docx

# Peer review: Decision Letter 1

**DOI:** 10.1371/journal.pone.0311391.r003

**Authors:** Tanay Chaubal

22 Aug 2024

PONE-D-23-42581R1Perception, preferences and practices of Endodontists and Restorative Dentists towards the use of dental magnification armamentarium- A multicentre studyPLOS ONE

Dear Dr. Iqbal,

Thank you for submitting your manuscript to PLOS ONE. After careful consideration, we feel that it has merit but does not fully meet PLOS ONE’s publication criteria as it currently stands. Therefore, we invite you to submit a revised version of the manuscript that addresses the points raised during the review process.

==============================

**ACADEMIC EDITOR: I congratulate on submitting an interesting manuscript. However, there are minor queries raised by the reviewers. I hope you could provide answers to the reviewers' queries.** **Thank you**

==============================

Please submit your revised manuscript by Oct 06 2024 11:59PM. If you will need more time than this to complete your revisions, please reply to this message or contact the journal office at plosone@plos.org. When you're ready to submit your revision, log on to https://www.editorialmanager.com/pone/ and select the 'Submissions Needing Revision' folder to locate your manuscript file.

Please include the following items when submitting your revised manuscript:

- A rebuttal letter that responds to each point raised by the academic editor and reviewer(s). You should upload this letter as a separate file labeled 'Response to Reviewers'.
- A marked-up copy of your manuscript that highlights changes made to the original version. You should upload this as a separate file labeled 'Revised Manuscript with Track Changes'.
- An unmarked version of your revised paper without tracked changes. You should upload this as a separate file labeled 'Manuscript'.

If you would like to make changes to your financial disclosure, please include your updated statement in your cover letter. Guidelines for resubmitting your figure files are available below the reviewer comments at the end of this letter.

If applicable, we recommend that you deposit your laboratory protocols in protocols.io to enhance the reproducibility of your results. Protocols.io assigns your protocol its own identifier (DOI) so that it can be cited independently in the future. For instructions see: https://journals.plos.org/plosone/s/submission-guidelines#loc-laboratory-protocols. Additionally, PLOS ONE offers an option for publishing peer-reviewed Lab Protocol articles, which describe protocols hosted on protocols.io. Read more information on sharing protocols at https://plos.org/protocols?utm_medium=editorial-email&utm_source=authorletters&utm_campaign=protocols.

We look forward to receiving your revised manuscript.

Kind regards,

Tanay Chaubal

Academic Editor

PLOS ONE

Journal Requirements:

Please review your reference list to ensure that it is complete and correct. If you have cited papers that have been retracted, please include the rationale for doing so in the manuscript text, or remove these references and replace them with relevant current references. Any changes to the reference list should be mentioned in the rebuttal letter that accompanies your revised manuscript. If you need to cite a retracted article, indicate the article’s retracted status in the References list and also include a citation and full reference for the retraction notice.

[Note: HTML markup is below. Please do not edit.]

Reviewers' comments:

Reviewer's Responses to Questions

**Comments to the Author**

1. If the authors have adequately addressed your comments raised in a previous round of review and you feel that this manuscript is now acceptable for publication, you may indicate that here to bypass the “Comments to the Author” section, enter your conflict of interest statement in the “Confidential to Editor” section, and submit your "Accept" recommendation.

Reviewer #3: (No Response)

Reviewer #4: All comments have been addressed

**********

2. Is the manuscript technically sound, and do the data support the conclusions?

The manuscript must describe a technically sound piece of scientific research with data that supports the conclusions. Experiments must have been conducted rigorously, with appropriate controls, replication, and sample sizes. The conclusions must be drawn appropriately based on the data presented. 

Reviewer #3: Yes

Reviewer #4: Yes

**********

3. Has the statistical analysis been performed appropriately and rigorously? 

Reviewer #3: Yes

Reviewer #4: I Don't Know

**********

4. Have the authors made all data underlying the findings in their manuscript fully available?

The PLOS Data policy requires authors to make all data underlying the findings described in their manuscript fully available without restriction, with rare exception (please refer to the Data Availability Statement in the manuscript PDF file). The data should be provided as part of the manuscript or its supporting information, or deposited to a public repository. For example, in addition to summary statistics, the data points behind means, medians and variance measures should be available. If there are restrictions on publicly sharing data—e.g. participant privacy or use of data from a third party—those must be specified.

Reviewer #3: Yes

Reviewer #4: Yes

**********

5. Is the manuscript presented in an intelligible fashion and written in standard English?

PLOS ONE does not copyedit accepted manuscripts, so the language in submitted articles must be clear, correct, and unambiguous. Any typographical or grammatical errors should be corrected at revision, so please note any specific errors here.

Reviewer #3: Yes

Reviewer #4: Yes

**********

6. Review Comments to the Author

Please use the space provided to explain your answers to the questions above. You may also include additional comments for the author, including concerns about dual publication, research ethics, or publication ethics. (Please upload your review as an attachment if it exceeds 20,000 characters)

Reviewer #3: The authors have adequately responded to the comments however the discussion requires more information. The authors should describe the strengths of their study as well as the implications of the results presented. That section can be made stronger and more impactful.

Reviewer #4: The authors are commended for a well written manuscript and the study will be of good interest to the readers.

Following the corrections made by the authors addressing most of the comments.

I will recommend considering the modification of the title to:Practices and preferences in the use of magnification among endodontists and restorative dentists - A multicentre study.

In addition, there is an abbreviations "it's" in line 276 that should be corrected.

A section that can be further improved is the conclusion, authors are advised to avoid the use of authoritative words such as "must". The last sentence can be rephrased in a way that subtle words like "requisite, obliged, required"

**********

7. PLOS authors have the option to publish the peer review history of their article (what does this mean?). If published, this will include your full peer review and any attached files.

If you choose “no”, your identity will remain anonymous but your review may still be made public.

**Do you want your identity to be public for this peer review?** For information about this choice, including consent withdrawal, please see our Privacy Policy.

Reviewer #3: No

Reviewer #4: No

**********

[NOTE: If reviewer comments were submitted as an attachment file, they will be attached to this email and accessible via the submission site. Please log into your account, locate the manuscript record, and check for the action link "View Attachments". If this link does not appear, there are no attachment files.]

While revising your submission, please upload your figure files to the Preflight Analysis and Conversion Engine (PACE) digital diagnostic tool, https://pacev2.apexcovantage.com/. PACE helps ensure that figures meet PLOS requirements. To use PACE, you must first register as a user. Registration is free. Then, login and navigate to the UPLOAD tab, where you will find detailed instructions on how to use the tool. If you encounter any issues or have any questions when using PACE, please email PLOS at figures@plos.org. Please note that Supporting Information files do not need this step.

# Author comment: Author response to Decision Letter 1

**DOI:** 10.1371/journal.pone.0311391.r004

25 Aug 2024

Comments:

Reviewer #3: 

Comment 1: The authors have adequately responded to the comments however the discussion requires more information. The authors should describe the strengths of their study as well as the implications of the results presented. That section can be made stronger and more impactful.

Authors Response: We appreciate the reviewer's insightful feedback and agree that the Discussion section could be further strengthened. We have revised the section to include a more comprehensive description of the study's strengths and the broader implications of our findings. 

Study Strengths: One of the key strengths of our study is its global, multicenter approach, which allows us to capture a wide range of perspectives from endodontists and restorative dentists across different regions. This diversity in sampling is crucial as it provides a more generalizable understanding of how dental magnification tools are perceived and utilized worldwide, rather than limiting the findings to a specific region or demographic. Moreover, our study focused on professionals with advanced degrees (master's and above), ensuring that the participants had substantial clinical experience and knowledge. This focus on well-trained practitioners increases the reliability of our findings, as their responses are informed by years of specialized practice rather than theoretical knowledge or limited experience. Additionally, by incorporating participants from both public and private sectors, we could compare and contrast the accessibility and usage of dental magnification tools in different practice settings, adding depth to our analysis.

Implications of the Results: The results of our study have significant implications for dental education, clinical practice, and policy-making. The strong endorsement of dental magnification devices as a standard of care by a majority of participants suggests that these tools are not only beneficial but potentially essential for modern dental practice. As a result, there is a clear need for dental education programs to integrate the use of these devices into their curricula, particularly at the postgraduate level. This integration will ensure that future dentists are well-equipped with the necessary skills to utilize magnification tools effectively, ultimately improving patient outcomes. Furthermore, our findings indicate a potential gap in access to magnification tools, particularly in certain regions and private practice settings. Addressing these disparities through policy changes, such as mandating the availability of these devices in all accredited dental programs and encouraging their use in private practices, could lead to more consistent and widespread adoption of these technologies. Finally, our study highlights the importance of ongoing professional development and training in the use of dental magnification devices. As demonstrated by the participants who acquired their skills through continuing education, lifelong learning is crucial for staying abreast of technological advancements in dentistry.

We believe that these additions provide a more robust and impactful discussion of our study.

Reviewer #4: 

The authors are commended for a well written manuscript and the study will be of good interest to the readers.

Following the corrections made by the authors addressing most of the comments.

Comment 1: I will recommend considering the modification of the title to: Practices and preferences in the use of magnification among endodontists and restorative dentists - A multicentre study.

Authors Response: We appreciate the positive feedback on our manuscript and thank you for the thoughtful suggestion regarding the title. We agree that the proposed title is more concise and focused, effectively capturing the essence of the study. Therefore, we have revised the title as follows:

Revised Title: "Practices and Preferences in the Use of Magnification Among Endodontists and Restorative Dentists: A Multicentre Study"

This modification aligns with the manuscript's content and enhances the clarity and impact of the title. Thank you for your valuable input.

Comment 2: In addition, there is an abbreviation "it's" in line 276 that should be corrected.

Authors Response: Thank you for bringing this to our attention. We have corrected the abbreviation "it’s" in line 300 to the appropriate form "it is" The sentence now accurately reflects the intended meaning without any grammatical errors. We appreciate your careful review and attention to detail.

Comment 3: A section that can be further improved is the conclusion, authors are advised to avoid the use of authoritative words such as "must". The last sentence can be rephrased in a way that subtle words like "requisite, obliged, required"

Authors Response: We appreciate the suggestion to refine the language in the conclusion to make it more balanced and less authoritative. We have revised the last sentence to incorporate more subtle wording, ensuring that the recommendation is still clear while being less directive.

Revised Conclusion: The perspective of the majority of participants in the current study was that dental magnification devices improve vision, ergonomics, quality of work, prevent eye fatigue, decrease operating time, improve prognosis, and thus should be considered the standard of care in modern endodontic and restorative dentistry practice. Therefore, it is recommended that the use of dental magnification devices be regarded as a requisite component of accredited postgraduate dental training programs and curricula.

This revision aligns with your feedback, creating a more measured and professional tone in our conclusion. Thank you for your valuable input.

Attachment Submitted filename: Response to Comments.docx

# Peer review: Decision Letter 2

**DOI:** 10.1371/journal.pone.0311391.r005

**Authors:** Tanay Chaubal

19 Sep 2024

Practices and Preferences in the Use of Magnification Among Endodontists and Restorative Dentists: A Multicentre Study

PONE-D-23-42581R2

Dear Dr. Azhar Iqbal,

We’re pleased to inform you that your manuscript has been judged scientifically suitable for publication and will be formally accepted for publication once it meets all outstanding technical requirements.

Within one week, you’ll receive an e-mail detailing the required amendments. When these have been addressed, you’ll receive a formal acceptance letter and your manuscript will be scheduled for publication.

An invoice will be generated when your article is formally accepted. Please note, if your institution has a publishing partnership with PLOS and your article meets the relevant criteria, all or part of your publication costs will be covered. Please make sure your user information is up-to-date by logging into Editorial Manager at Editorial Manager® and clicking the ‘Update My Information' link at the top of the page. If you have any questions relating to publication charges, please contact our Author Billing department directly at authorbilling@plos.org.

If your institution or institutions have a press office, please notify them about your upcoming paper to help maximize its impact. If they’ll be preparing press materials, please inform our press team as soon as possible -- no later than 48 hours after receiving the formal acceptance. Your manuscript will remain under strict press embargo until 2 pm Eastern Time on the date of publication. For more information, please contact onepress@plos.org.

Kind regards,

Tanay Chaubal

Academic Editor

PLOS ONE

Additional Editor Comments (optional):

Reviewers' comments:

Reviewer's Responses to Questions

**Comments to the Author**

1. If the authors have adequately addressed your comments raised in a previous round of review and you feel that this manuscript is now acceptable for publication, you may indicate that here to bypass the “Comments to the Author” section, enter your conflict of interest statement in the “Confidential to Editor” section, and submit your "Accept" recommendation.

Reviewer #3: All comments have been addressed

Reviewer #4: All comments have been addressed

**********

2. Is the manuscript technically sound, and do the data support the conclusions?

The manuscript must describe a technically sound piece of scientific research with data that supports the conclusions. Experiments must have been conducted rigorously, with appropriate controls, replication, and sample sizes. The conclusions must be drawn appropriately based on the data presented. 

Reviewer #3: Yes

Reviewer #4: Yes

**********

3. Has the statistical analysis been performed appropriately and rigorously? 

Reviewer #3: Yes

Reviewer #4: Yes

**********

4. Have the authors made all data underlying the findings in their manuscript fully available?

The PLOS Data policy requires authors to make all data underlying the findings described in their manuscript fully available without restriction, with rare exception (please refer to the Data Availability Statement in the manuscript PDF file). The data should be provided as part of the manuscript or its supporting information, or deposited to a public repository. For example, in addition to summary statistics, the data points behind means, medians and variance measures should be available. If there are restrictions on publicly sharing data—e.g. participant privacy or use of data from a third party—those must be specified.

Reviewer #3: Yes

Reviewer #4: Yes

**********

5. Is the manuscript presented in an intelligible fashion and written in standard English?

PLOS ONE does not copyedit accepted manuscripts, so the language in submitted articles must be clear, correct, and unambiguous. Any typographical or grammatical errors should be corrected at revision, so please note any specific errors here.

Reviewer #3: Yes

Reviewer #4: Yes

**********

6. Review Comments to the Author

Please use the space provided to explain your answers to the questions above. You may also include additional comments for the author, including concerns about dual publication, research ethics, or publication ethics. (Please upload your review as an attachment if it exceeds 20,000 characters)

Reviewer #3: The authors have addressed my previous concerns adequately, the manuscript is very well-written and will be interesting to the readership.

Reviewer #4: (No Response)

**********

7. PLOS authors have the option to publish the peer review history of their article (what does this mean?). If published, this will include your full peer review and any attached files.

If you choose “no”, your identity will remain anonymous but your review may still be made public.

**Do you want your identity to be public for this peer review?** For information about this choice, including consent withdrawal, please see our Privacy Policy.

Reviewer #3: No

Reviewer #4: No

**********

# Editor report: Acceptance letter

**DOI:** 10.1371/journal.pone.0311391.r006

**Authors:** Tanay Chaubal

7 Nov 2024

PONE-D-23-42581R2 

PLOS ONE

Dear Dr. Iqbal, 

I'm pleased to inform you that your manuscript has been deemed suitable for publication in PLOS ONE. Congratulations! Your manuscript is now being handed over to our production team.

At this stage, our production department will prepare your paper for publication. This includes ensuring the following:

* All references, tables, and figures are properly cited

* All relevant supporting information is included in the manuscript submission,

* There are no issues that prevent the paper from being properly typeset

If revisions are needed, the production department will contact you directly to resolve them. If no revisions are needed, you will receive an email when the publication date has been set. At this time, we do not offer pre-publication proofs to authors during production of the accepted work. Please keep in mind that we are working through a large volume of accepted articles, so please give us a few weeks to review your paper and let you know the next and final steps. 

Lastly, if your institution or institutions have a press office, please let them know about your upcoming paper now to help maximize its impact. If they'll be preparing press materials, please inform our press team within the next 48 hours. Your manuscript will remain under strict press embargo until 2 pm Eastern Time on the date of publication. For more information, please contact onepress@plos.org.

If we can help with anything else, please email us at customercare@plos.org.

Thank you for submitting your work to PLOS ONE and supporting open access. 

Kind regards, 

PLOS ONE Editorial Office Staff

on behalf of

Dr. Tanay Chaubal 

Academic Editor

PLOS ONE
